# Clinical Characteristics and Prognostic Factors of Early and Late Recurrence After Definitive Radiotherapy for Nasopharyngeal Carcinoma

**DOI:** 10.3389/fonc.2020.01469

**Published:** 2020-08-25

**Authors:** Feng Li, Fo-Ping Chen, Yu-Pei Chen, Yue Chen, Xiao-Jun He, Xiao-Dan Huang, Zi-Qi Zheng, Wei-Hong Zheng, Xu Liu, Ying Sun, Guan-Qun Zhou

**Affiliations:** ^1^Department of Radiation Oncology, Sun Yat-sen University Cancer Center, Guangzhou, China; ^2^State Key Laboratory of Oncology in South China, Sun Yat-sen University Cancer Center, Guangzhou, China; ^3^Collaborative Innovation Center of Cancer Medicine, Sun Yat-sen University Cancer Center, Guangzhou, China; ^4^Guangdong Key Laboratory of Nasopharyngeal Carcinoma Diagnosis and Therapy, Sun Yat-sen University Cancer Center, Guangzhou, China

**Keywords:** nasopharyngeal carcinoma, radiotherapy, prognosis, early locoregional recurrence, late locoregional recurrence

## Abstract

We investigated the clinical characteristics, prognostic factors, and post-recurrence prognostic factors of early- and late-recurrence patients for nasopharyngeal carcinoma (NPC) after definitive intensity-modulated radiation therapy (IMRT). This was a single-center retrospective analysis of patients in China from January 2010 to December 2015. The prognostic factors for overall survival (OS) and post-recurrence OS of early- and late-recurrence patients were identified using univariate and multivariate Cox regression analyses. Of the 9,468 patients included, 409 (4.3%), 325 (3.4%), and 182(1.9%) developed purely local recurrence, purely regional recurrence, and locoregional recurrence during follow-up, respectively. In the purely local recurrence group, 192 patients (46.9%) developed early local recurrence (ETR), and 217 patients (53.1%) developed late local recurrence (LTR). Of the 192 ETR patients, multivariate Cox regression analysis revealed that age and gender were independent risk factors of OS, and post-recurrence best supportive treatment (PRBST) was associated with poorer post-recurrence OS. Of the 217 LTR patients, the results revealed that baseline value of EBV-DNA was an independent risk factor for OS, while PRBST was associated with poorer post-recurrence OS. In the purely regional recurrence group, 183 patients (56.3%) developed early regional recurrence (ENR), and 142 patients (43.7%) developed late regional recurrence (LNR). Of the 183 ENR patients, multivariate Cox regression analysis revealed that alcohol abuse and TNM stage were independent risk factors of OS, while alcohol drinkers and PRBST were associated with poorer post-recurrence OS. Of the 142 LNR patients, PRBST was associated with poorer post-recurrence OS. In the locoregional recurrence group, 87 patients (47.8%) developed early locoregional recurrence (ELR), and 95 patients (52.2%) developed late locoregional recurrence (LLR). Of the 87 ELR patients, multivariate Cox regression analysis revealed that N stage and TNM stage were independent risk factors of OS, and N2/3 stage and PRBST were associated with poorer post-recurrence OS. Of the 95 LLR patients, the results revealed that T stage was an independent risk factor for OS, while T3/4 stage and PRBST were associated with poorer post-recurrence OS. Patients with LTR/LNR/LLR demonstrate significantly better OS compared with patients with ETR/ENR/ELR, Nevertheless, post-recurrence OS between patients with ETR/ENR/ELR and LTR/LNR/LLR was not significantly different.

## Introduction

Nasopharyngeal carcinoma (NPC), a malignant tumor that originates in the nasopharyngeal epithelium, is endemic in Southern China, Southeast Asia, North Africa, the Middle East, and Alaska (1, 2). As a result of its complex anatomical location and high radiosensitivity, radiotherapy with or without chemotherapy is the primary treatment modality for NPC (3, 4), and the application of IMRT has greatly improved locoregional control in NPC (5). However, the long-term prognosis remains unsatisfactory, given the high rate of locoregional recurrence of up to 5–10% in patients after definitive IMRT (6). This study focuses on the failure patterns of NPC except distant metastasis, which was separated clearly in three subgroups: (1) purely local recurrence (on the T site only), (2) purely regional recurrence (on the N site only), (3) locoregional recurrence (on the T and N sites simultaneously). Meanwhile, time to cancer recurrence differs in such patients, and the three subgroups were divided into ETR and LTR, ENR and LNR, and ELR and LLR, respectively, based on the time to recurrence after radiotherapy (7–9). To the best of our knowledge, research focusing on early and late recurrence in NPC patients remains rare and limited. Accordingly, we aimed to identify the clinical characteristics and prognostic factors of ETR and LTR, ENR and LNR, and ELR and LLR in a large cohort of patients with NPC who underwent long-term follow-up, providing data to clinicians for planning surveillance strategies.

## Patients and Methods

### Patient Selection

This study was performed according to the ethical principles of the Declaration of Helsinki, and the Sun Yat-sen University Cancer Center review board approved the study protocol. Written informed consent was obtained from all patients for their data to be used in clinical research without affecting their treatment options or violating their privacy. We retrospectively reviewed the records of all 9,468 patients with biopsy-proven NPC who had been treated with IMRT at our center between January 2010 and December 2015. All patients had completed a pretreatment evaluation including complete patient history, physical examination, hematology and biochemistry profiles, nasopharynx and neck magnetic resonance imaging (MRI), chest radiography, abdominal ultrasonography, and whole-body bone scan or positron emission tomography/computed tomography (PET/CT). All patients were restaged according to the 8th Union for International Cancer Control/American Joint Committee on Cancer staging system (10, 11). RT+Chemo was defined as treatment with both radiotherapy and chemotherapy, including induction chemotherapy and/or concurrent chemotherapy and/or adjuvant chemotherapy. Treatment options after recurrence were divided into four parts: salvage surgery, re-irradiation, chemotherapy, and best supportive treatment. During the study period, our institutional guidelines recommended no chemotherapy for stage I–IIA NPC, concurrent chemoradiation therapy for stage IIB NPC, and concurrent chemoradiation therapy with or without neoadjuvant/adjuvant chemotherapy for stage III to IVA–B NPC.

### Follow-Up Schedule and Definition of ETR and LTR, ENR and LNR, and ELR and LLR

Patients attended follow-up visits every 3 months during the first 2 years, every 6 months during years 3–5, and annually thereafter or until death. Scheduled surveillance including fiberoptic endoscopy and head and neck CT/MRI scans was performed every 3 months during the first year and annually during years 2–5. Local recurrence was diagnosed by fiberoptic endoscopy and biopsy or nasopharynx and skull base CT/MRI scans. Regional recurrence was diagnosed by pathological examination with fine-needle aspiration or surgery or by radiology with neck CT/MRI scans. Additional tests were ordered whenever necessary. In this study, purely local recurrence was defined as recurrence on the T site only, which was divided into ETR and LTR according to time to NPC recurrence of ≤2 years and >2 years. Purely regional recurrence was defined as recurrence on the N site only, which was divided into ENR and LNR according to time to NPC recurrence of ≤2 years and >2 years, and locoregional recurrence was defined as recurrence on the T and N sites simultaneously, which was divided into ELR and LLR according to the time to NPC recurrence of ≤2 years and >2 years.

### Statistical Analysis

The patients' clinical and pathological characteristics were summarized using frequencies and percentages for categorical covariates and medians and ranges for continuous covariates. The clinicopathological characteristics and treatment modalities among the patients with ETR and LTR, ENR and LNR, and ELR and LLR were compared using the chi-square test. The OS and post-recurrence OS were calculated with the Kaplan–Meier method, and differences between survival curves were assessed with the log-rank test. The prognostic factors of OS and post-recurrence OS of the patients with ETR and LTR, ENR and LNR, and ELR and LLR were evaluated using multivariate Cox regression analysis. *P* < 0.05 was considered significant. Statistical analyses were performed using SPSS version 23.0 (IBM).

## Results

### Comparison of Clinical Characteristics Between Patients With ETR and LTR, ENR and LNR, and ELR and LLR

Of the 9,468 patients included, 409 (4.3%) developed purely local recurrence, 325 (3.4%) developed purely regional recurrence, and 182 (1.9%) developed locoregional recurrence. Among the 409 patients with purely local recurrence, in whom the median time to recurrence was 25.4 months (range, 3.7–86.3 months), 207 patients (50.6%) died, 303 patients (74.1%) were male, 106 patients (25.9%) were female, and the median age was 47.0 years. At a median follow-up of 44.5 months (range, 9.9–104.9 months), 192 patients (46.9%) developed ETR, with a median time to recurrence of 15.4 months (range, 3.7–24.0 months); 217 patients (53.1%) developed LTR, with a median time to recurrence of 36.7 months (range, 24.1–86.3 months). After recurrence, 47 patients (11.5%) received BST, 19 patents (4.7%) were undergoing salvage surgery, 203 patients (49.6%) received re-irradiation, and 140 patients (34.2%) received chemotherapy. Among the 325 patients with purely regional recurrence, 114 patients (35.1%) died, 261 patients (80.3%) were male, 64 patients (19.7%) were female, and the median age was 45.0 years. At a median follow-up of 49.3 months (range, 7.9–111.0 months), 183 patients (56.3%) developed ENR, with a median time to recurrence of 14.5 months (range, 1.8–23.9 months), and 142 patients (43.7%) developed LNR, with a median time to recurrence of 37.5 months (range, 24.4–80.1 months). Among the 182 patients with locoregional recurrence, 88 patients (48.4%) died, 138 patients (75.8%) were male, 44 patients (24.2%) were female, and the median age was 44.0 years. At a median follow-up of 49.9 months (range, 6.9–101.8 months), 87 patients (47.8%) developed ELR, with a median time to recurrence of 14.70 months (range, 4.90–23.63 months), and 95 patients (52.2%) developed LLR, with a median time to recurrence of 34.63 months (range, 24.07–94.13 months). Tables 1–3 illustrate the comparisons of the baseline clinical characteristics between the patients with ETR and LTR, ENR and LNR, and ELR and LLR. The difference was significant in the baseline value of EBV-DNA between ENR and LNR groups (*P* = 0.009). No significant differences were found in the clinicopathological characteristics between ETR and LTR, ENR and LNR, and ELR and LLR.

**Table 1:** Comparison of clinical characteristics of ETR and LTR patients in the purely local recurrence group.

**Characteristic**	**Total**	**ETR group**	**LTR group**	***P*-value**
	**(*n* = 409)%**	**(*n* = 192)%**	**(*n* = 217)%**	
**Age (years)**				0.203
≤46 years	199 (48.7)	87 (45.3)	112 (51.6)	
>46 years	210 (51.3)	105 (54.7)	105 (48.4)	
**Gender**				0.282
Male	303 (74.1)	147 (76.6)	156 (71.9)	
Female	106 (25.9)	45 (23.4)	61 (28.1)	
**Smoking status**				0.535
Non-smoker	243 (59.4)	111 (57.8)	132 (60.8)	
Smoker	166 (40.6)	81 (42.2)	85 (39.2)	
**Alcohol abuse**				0.804
Non-drinker	368 (90.0)	172 (89.6)	196 (90.3)	
Drinker	41 (10.0)	20 (10.4)	21 (9.7)	
**Tumor family history**				0.410
No	308 (75.3)	141 (73.4)	167 (77.0)	
Yes	101 (24.7)	51 (26.6)	50 (23.0)	
**Cranial nerve symptom**				0.741
N0	356 (87.0)	166 (86.5)	190 (87.6)	
Yes	53 (13.0)	26 (13.5)	27 (12.4)	
**Baseline value of EBV-DNA**				0.212
≤2,000	189 (46.2)	95 (49.5)	94 (43.3)	
>2,000	220 (53.8)	97 (50.5)	123 (56.7)	
**Histological type**				0.218
WHO I/II	18 (4.4)	11 (5.7)	7 (3.2)	
WHO III	391 (95.6)	181 (94.3)	210 (96.8)	
**T stage**				0.084
1/2	60 (14.7)	22 (11.5)	38 (17.5)	
3/4	349 (85.3)	170 (88.5)	179 (82.5)	
**N stage**				0.258
0/1	274 (67.0)	134 (69.8)	140 (64.5)	
2/3	135 (33.0)	58 (30.2)	77 (35.5)	
**TNM stage**				0.416
I/II	46 (11.2)	19 (9.9)	27 (12.4)	
III/IV	363 (88.8)	173 (90.1)	190 (87.6)	
**Induction chemotherapy**				0.927
No	195 (47.7)	92 (47.9)	103 (47.5)	
Yes	214 (52.3)	100 (52.1)	252 (52.5)	
**Concurrent chemotherapy**				0.171
No	86 (21.0)	46 (24.0)	40 (18.4)	
Yes	323 (79.0)	146 (76.0)	17781.6)	
**Adjuvant chemotherapy**				0.404
No	388 (94.9)	184 (95.8)	204 (94.0)	
Yes	21 (5.1)	8 (4.2)	13 (6.0)	
**Post-recurrence treatment options**				0.073
BST	47 (11.5)	26 (13.5)	21 (9.7)	
Salvage surgery	19 (4.7)	8 (4.2)	11 (5.1)	
Re-irradiation	203 (49.6)	83 (43.2)	120 (55.3)	
Chemotherapy	140 (34.2)	75 (39.1)	65 (29.9)	

**Table 2:** Comparison of clinical characteristics of ENR and LNR patients in the purely regional recurrence group.

**Characteristic**	**Total**	**ENR group**	**LNR group**	***P*-value**
	**(*n* = 325)%**	**(*n* = 183)%**	**(*n* = 142)%**	
**Age (years)**				0.094
≤46 years	175 (53.8)	106 (57.9)	69 (48.6)	
>46 years	150 (46.2)	77 (42.1)	73 (51.4)	
**Gender**				0.567
Male	261 (80.3)	149 (81.4)	112 (78.9)	
Female	64 (19.7)	34 (18.6)	30 (21.1)	
**Smoking status**				0.165
Non-smoker	192 (59.1)	102 (55.7)	90 (63.4)	
Smoker	133 (40.9)	81 (44.3)	52 (36.6)	
**Alcohol abuse**				0.420
Non-drinker	266 (81.8)	147 (80.3)	119 (83.8)	
Drinker	59 (18.2)	36 (19.7)	23 (16.2)	
**Tumor family history**				0.796
No	229 (70.5)	130 (71.0)	99 (69.7)	
Yes	96 (29.5)	53 (29.0)	43 (30.3)	
**Cranial nerve symptom**				0.936
N0	304 (93.5)	171 (93.4)	133 (93.7)	
Yes	21 (6.5)	12 (6.6)	9 (6.3)	
**Baseline value of EBV-DNA**				0.009
≤2,000	84 (25.8)	37 (20.2)	47 (33.1)	
>2,000	241 (74.2)	146 (79.8)	95 (66.9)	
**Histological type**				0.183
WHO I/II	12 (3.7)	9 (4.9)	3 (2.1)	
WHO III	313 (96.3)	174 (95.1)	139 (97.9)	
**T stage**				0.337
1/2	117 (36.0)	70 (38.3)	47 (33.1)	
3/4	208 (64.0)	113 (61.7)	95 (66.9)	
**N stage**				0.157
0/1	139 (42.8)	72 (39.3)	67 (47.2)	
2/3	186 (57.2)	111 (60.7)	75 (52.8)	
**TNM stage**				0.606
I/II	59 (18.2)	35 (19.1)	24 (16.9)	
III/IV	266 (81.8)	148 (80.9)	118 (83.1)	
**Induction chemotherapy**				0.077
No	111 (34.2)	55 (30.1)	56 (39.4)	
Yes	214 (65.8)	128 (69.9)	86 (60.6)	
**Concurrent chemotherapy**				0.625
No	47 (14.5)	28 (15.3)	19 (13.4)	
Yes	278 (85.5)	155 (84.7)	123 (86.6)	
**Adjuvant chemotherapy**				0.113
No	295 (90.8)	162 (88.5)	133 (93.7)	
Yes	30 (9.2)	21 (11.5)	9 (6.3)	
**Post-recurrence treatment options**				0.689
BST	17 (5.2)	9 (4.9)	8 (5.6)	
Salvage surgery	162 (49.9)	94 (51.4)	68 (47.9)	
Re-irradiation	64 (19.7)	32 (17.5)	32 (22.5)	
Chemotherapy	82 (25.2)	48 (26.2)	34 (24.0)	

**Table 3:** Comparison of clinical characteristics of ELR and LLR patients in the locoregional recurrence group.

**Characteristic**	**Total**	**ELR group**	**LLR group**	***P*-value**
	**(*n* = 182)%**	**(*n* = 87)%**	**(*n* = 95)%**	
**Age (years)**				0.088
≤46 years	101 (55.5)	54 (62.1)	47 (49.5)	
>46 years	81 (44.5)	33 (37.9)	48 (50.5)	
**Gender**				0.162
Male	138 (75.8)	70 (80.5)	68 (71.6)	
Female	44 (24.2)	17 (19.5)	27 (28.4)	
**Smoking status**				0.870
Non-smoker	112 (61.5)	53 (60.9)	59 (62.1)	
Smoker	70 (38.5)	34 (39.1)	36 (37.9)	
**Alcohol abuse**				0.569
Non-drinker	154 (84.6)	75 (86.2)	79 (83.2)	
Drinker	28 (15.4)	12 (13.8)	16 (16.8)	
**Tumor family history**				0.603
No	137 (75.3)	67 (77.0)	70 (73.7)	
Yes	45 (24.7)	20 (23.0)	25 (26.3)	
**Cranial nerve symptom**				0.427
N0	172 (94.5)	81 (93.1)	91 (95.8)	
Yes	10 (5.5)	6 (6.9)	4 (4.2)	
**Baseline value of EBV-DNA**				0.728
≤2,000	63 (34.6)	29 (33.3)	34 (35.8)	
>2,000	119 (65.4)	58 (66.7)	61 (64.2)	
**Histological type**				0.246
WHO I/II	10 (5.5)	3 (3.4)	7 (7.4)	
WHO III	172 (94.5)	84 (96.6)	88 (92.6)	
**T stage**				0.552
1/2	39 (21.4)	17 (19.5)	22 (23.2)	
3/4	143 (78.6)	70 (80.5)	73 (76.8)	
**N stage**				0.316
0/1	97 (53.3)	43 (49.4)	54 (56.8)	
2/3	85 (46.7)	44 (50.6)	41 (43.2)	
**TNM stage**				0.826
I/II	22 (12.1)	11 (12.6)	11 (11.6)	
III/IV	160 (87.9)	76 (87.4)	84 (88.4)	
**Induction chemotherapy**				0.399
No	77 (42.3)	34 (39.1)	43 (45.3)	
Yes	105 (57.7)	53 (60.9)	52 (54.7)	
**Concurrent chemotherapy**				0.203
No	29 (15.9)	17 (19.5)	12 (12.6)	
Yes	153 (84.1)	70 (80.5)	83 (87.4)	
**Adjuvant chemotherapy**				0.065
No	173 (95.1)	80 (92.0)	93 (97.9)	
Yes	9 (4.9)	7 (8.0)	2 (2.1)	
**Post-recurrence treatment options**				0.415
BST	9 (4.9)	5 (5.7)	4 (4.2)	
Salvage surgery	43 (23.6)	22 (25.3)	21 (22.1)	
Re-irradiation	70 (38.5)	28 (32.2)	42 (44.2)	
Chemotherapy	60 (33.0)	32 (36.8)	28 (29.5)	

### Prognostic Factors Associated With OS

**Figure 1:**
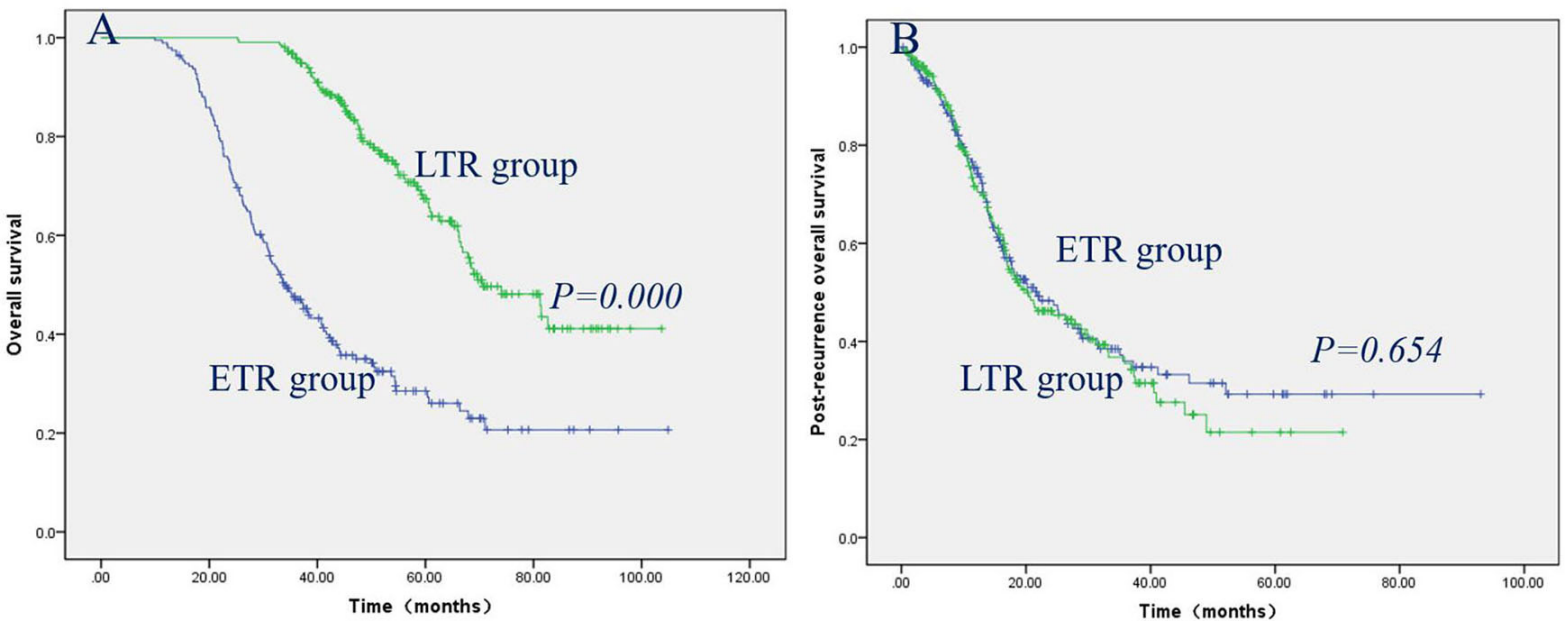
Patients with LTR had significantly better OS than patients with ETR **(A)**, while post-recurrence OS did not reach significance between the patients with LTR and ETR **(B)**.

**Figure 2:**
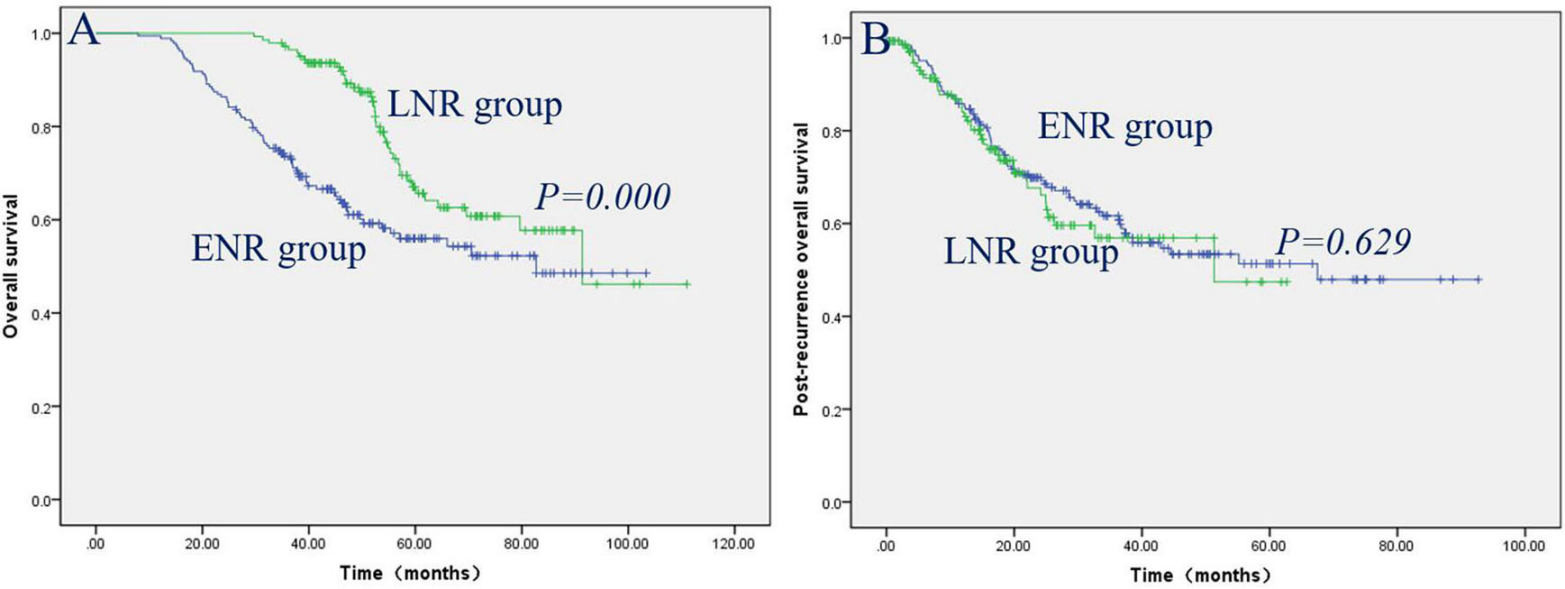
Patients with LNR had significantly better OS than patients with ENR **(A)**, while post-recurrence OS did not reach significance between the patients with LNR and ENR **(B)**.

**Figure 3:**
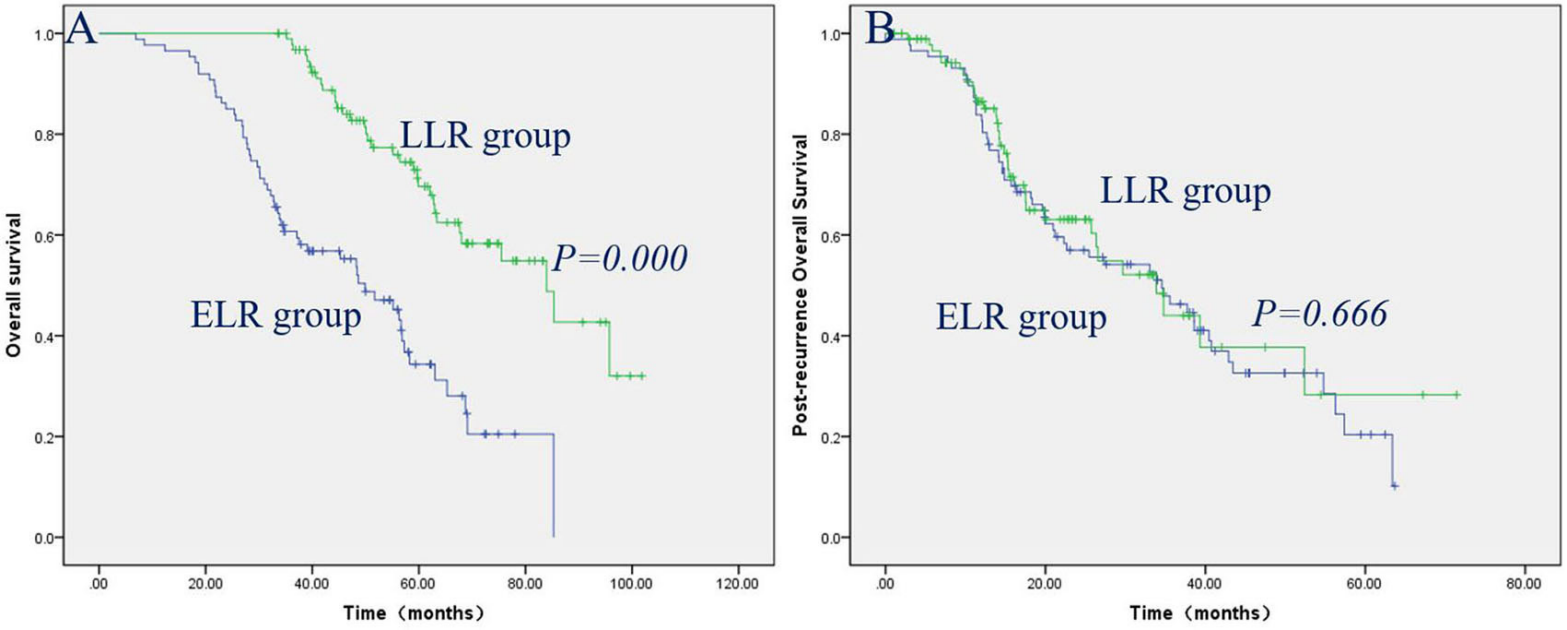
Patients with LLR had significantly better OS than patients with ELR **(A)**, while post-recurrence OS did not reach significance between the patients with LLR and ELR **(B)**.

The prognostic factors contributing to long-term OS in ETR and LTR, ENR and LNR, and ELR and LLR were investigated using univariate and multivariate analyses (Tables 4–6). The effects of clinical factors on the OS with ETR group were evaluated. Age > 46 years and male gender were significantly associated with poorer OS. Cox regression modeling predicted that age [hazard ratio [HR], 1.645; 95% confidence interval [CI], 1.153–2.346; *P* = 0.006], gender (HR, 0.589; 95% CI, 0.377–0.920; *P* = 0.020) were independent risk factors of OS. Of the 217 patients with LTR, a baseline value of EBV-DNA > 2,000 was significantly associated with poorer OS. Cox regression modeling identified the baseline value of EBV-DNA (HR, 1.817; 95% CI, 1.115–2.962; *P* = 0.017) as an independent risk factor of OS. The effects of clinical factors on the OS with ENR group were evaluated. Alcohol drinking and TNM stage III/IV were significantly associated with poorer OS. Cox regression modeling predicted that alcohol abuse [hazard ratio [HR], 3.070; 95% confidence interval [CI], 1.551–6.076; *P* = 0.001], TNM stage (HR, 2.394; 95% CI, 1.178–4.864; *P* = 0.016) were independent risk factors of OS. Of the 142 patients with LNR, no clinical characteristics were significantly associated with OS. The effects of clinical factors on the OS with ELR group were also evaluated. Cox regression modeling predicted that N stage [hazard ratio [HR], 2.391; 95% confidence interval [CI], 1.328–4.271; *P* = 0.004], TNM stage (HR, 1.874; 95% CI, 1.248–2.812; *P* = 0.002) were independent risk factors of OS. Of the 95 patients with LLR, Cox regression modeling identified T stage (HR, 3.675; 95% CI, 1.241–10.882; *P* = 0.019) as an independent risk factor of OS. The patients with LTR/LNR/LLR demonstrated significantly better OS than the patients with ETR/ENR/ELR (Figures 1A, 2A, 3A), with a median OS of 33.1 months (range, 9.9–104.9 months)/53.0 months (range, 25.2–103.7 months), 44.1 months (range, 7.9–103.3 months)/53.6 months (range, 29.6–111.0 months), and 39.10 months (range, 6.90–85.27 months)/58.9 months (range, 33.6–101.8 months), respectively.

**Table 4:** Univariate and multivariate analysis of prognostic factors of ETR and LTR in the purely local recurrence group.

**Characteristic**	**ETR group**			**LTR group**		
	**Univariate** ***P*****-value HR (95% CI)**		**Multivariate *P*-value**	**Univariate** ***P*****-value HR (95% CI)**		**Multivariate *P*-value**
**Age (years)**	0.005		0.006	0.888		NS
≤46 years		Reference				
>46 years		1.645 (1.153–2.346)			/	
**Gender**	0.008		0.020	0.179		NS
Male		Reference				
Female		0.589 (0.377–0.920)			/	
**Smoking status**	0.293		NS	0.264		NS
Non-smoker						
Smoker		/			/	
**Alcohol abuse**	0.211		NS	0.043		NS
Non-drinker						
Drinker		/			/	
**Tumor family history**	0.883		NS	0.079		NS
No						
Yes		/			/	
**Cranial nerve symptom**	0.025		NS	0.836		NS
No						
Yes		/			/	
**Baseline value of EBV-DNA**	0.381		NS	0.015		0.017
≤2000					Reference	
>2000		/			1.817 (1.115–2.962)	
**Histological type**	0.257		NS	0.399		NS
WHO I/II						
WHO III		/			/	
**T stage**	0.110		NS	0.184		NS
1/2						
3/4		/			/	
**N stage**	0.074		NS	0.150		NS
0/1						
2/3		/			/	
**TNM stage**	0.069		NS	0.479		NS
I/II						
III/IV					/	
**RT+/Chemo**	0.316		NS	0.182		NS
RT alone						
RT+Chemo		/			/	

**Table 5:** Univariate and multivariate analysis of prognostic factors of ENR and LNR in the purely regional recurrence group.

**Characteristic**	**ETR group**			**LTR group**		
	**Univariate** ***P*****-value HR (95% CI)**		**Multivariate *P*-value**	**Univariate** ***P*****-value HR (95% CI)**		**Multivariate *P*-value**
**Age (years)**	0.437		NS	0.997		NS
≤46 years						
>46 years		/			/	
**Gender**	0.749		NS	0.885		NS
Male						
Female		/			/	
**Smoking status**	0.264		NS	0.705		NS
Non-smoker						
Smoker		/			/	
**Alcohol abuse**	0.032		0.001	0.970		NS
Non-drinker		Reference				
Drinker		3.070 (1.551–6.076)			/	
**Tumor family history**	0.599		NS	0.796		NS
No						
Yes		/			/	
**Cranial nerve symptom**	0.035		NS	0.315		NS
No						
Yes		/			/	
**Baseline value of EBV-DNA**	0.090		NS	0.323	/	NS
≤2,000						
>2,000		/			/	
**Histological type**	0.979		NS	0.234		NS
WHO I/II						
WHO III		/			/	
**T stage**	0.353		NS	0.580		NS
1/2						
3/4		/			/	
**N stage**	0.048		NS	0.192		NS
0/1						
2/3		/			/	
**TNM stage**	0.037		0.016	0.494		NS
I/II		Reference				
III/IV		2.394 (1.178–4.864)			/	
**RT+/Chemo**	0.823		NS	0.740		NS
RT alone						
RT+Chemo		/			/	

**Table 6:** Univariate and multivariate analysis of prognostic factors of ELR and LLR in the locoregional recurrence group.

**Characteristic**	**ETR group**			**LTR group**		
	**Univariate** ***P*****-value HR (95% CI)**		**Multivariate *P*-value**	**Univariate** ***P*****-value HR (95% CI)**		**Multivariate *P*-value**
**Age(years)**	0.069		NS	0.063		NS
≤46 years						
>46 years		/			/	
**Gender**	0.837		NS	0.101		NS
Male						
Female		/			/	
**Smoking status**	0.245		NS	0.483		NS
Non-smoker						
Smoker		/			/	
**Alcohol abuse**	0.194		NS	0.817		NS
Non-drinker						
Drinker		/			/	
**Tumor family history**	0.106		NS	0.095		NS
No						
Yes		/			/	
**Cranial nerve symptom**	0.139		NS	0.771		NS
No						
Yes		/			/	
**Baseline value of EBV-DNA**	0.110		NS	0.600		NS
≤2,000						
>2,000		/			/	
**Histological type**	0.646		NS	0.392		NS
WHO I/II						
WHO III		/			/	
**T stage**	0.557		NS	0.020		0.019
1/2					Reference	
3/4		/			3.675 (1.241–10.882)	
**N stage**	0.026		0.004	0.966		NS
0/1		Reference				
2/3		2.391 (1.328–4.271)			/	
**TNM stage**	0.363		0.002	0.047		NS
I/II		Reference				
III/IV		1.874 (1.248–2.812)			/	
**RT+/Chemo**	0.765		NS	0.403		NS
RT alone						
RT+Chemo		/			/	

### Prognostic Factors Associated With Post-recurrence OS

The clinical factors and treatment modalities of post-recurrence OS in ETR and LTR, ENR and LNR, and ELR and LLR were elevated by univariate and multivariate analyses (Tables 7–9). Of the 192 patients with ETR, PRBST was significantly associated with poorer OS. Of the 217 patients with LTR patients, Cox regression modeling identified post-recurrence treatment options (*P* = 0.000) was an independent risk factor of post-recurrence OS. Of the 183 patients with ENR, Cox regression modeling predicted that alcohol abuse (HR, 3.750; 95% CI, 1.909–7.367; *P* = 0.000) and post-recurrence treatment options (*P* = 0.000) were independent risk factors of post-recurrence OS. Of the 142 patients with LNR patients, Cox regression modeling predicted post-recurrence treatment options (*P* = 0.000) was an independent risk factor of post-recurrence OS. Of the 87 patients with ELR, Cox regression modeling predicted that N stage (HR, 2.216; 95% CI, 1.225−4.009; *P* = 0.008) and post-recurrence treatment options (*P* = 0.000) were independent risk factors of post-recurrence OS. Of the 95 patients with LLR patients, Cox regression modeling predicted that T stage (HR, 4.111; 95% CI, 1.337–12.635; *P* = 0.014) and post-recurrence treatment options (*P* = 0.000) were independent risk factors of post-recurrence OS. Post-recurrence OS was not significantly different between ETR and LTR, ENR and LNR, and ELR and LLR groups (Figures 1B, 2B, 3B), with a median post-recurrence OS of 16.2 months (range, 0–93.0 months) and 12.2 months (range, 0.2–69.1 months), 28.1 months (range, 0.5–92.6 months) and 15.9 months (range 0–62.6 months), and 22.6 months (range, 0–63.7 months) and 15.3 months (range, 0.6–71.4 months), respectively.

**Table 7:** Univariate and multivariate analysis of post-recurrence prognostic factors of ETR and LTR in the purely local recurrence group.

**Characteristic**	**ETR group**			**LTR group**		
	**Univariate** ***P*****-value HR (95% CI)**		**Multivariate *P*-value**	**Univariate** ***P*****-value HR (95% CI)**		**Multivariate *P*-value**
**Age (years)**	0.013		NS	0.144		NS
≤46 years						
>46 years		/			/	
**Gender**	0.014		NS	0.299		NS
Male						
Female		/			/	
**Smoking status**	0.386		NS	0.262		NS
Non-smoker						
Smoker		/			/	
**Alcohol abuse**	0.296		NS	0.038		NS
Non-drinker						
Drinker		/			/	
**Tumor family history**	0.786		NS	0.077		NS
No						
Yes		/			/	
**Cranial nerve symptom**	0.061	/	NS	0.994		NS
No						
Yes		/			/	
**Baseline value of EBV-DNA**	0.299		NS	0.055		NS
≤2,000						
>2,000		/			/	
**Histological type**	0.296		NS	0.794		NS
WHO I/II						
WHO III		/			/	
**T stage**	0.124		NS	0.297		NS
1/2						
3/4		/			/	
**N stage**	0.095		NS	0.486		NS
0/1						
2/3		/			/	
**TNM stage**	0.095		NS	0.669		NS
I/II						
III/IV		/				
**Post-recurrence treatment options**	0.000		0.000	0.007	/	0.019
BST		Reference			Reference	
Salvage surgery		0.162 (0.065–0.407)			0.983 (0.359–2.691)	
Re-irradiation		0.226 (0.135–0.378)			0.369 (0.184–0.739)	
Chemotherapy		0.302 (0.184–0.497)			0.554 (0.270–1.138)	

**Table 8:** Univariate and multivariate analysis of post-recurrence prognostic factors of ENR and LNR in the purely regional recurrence group.

**Characteristic**	**ETR group**			**LTR group**		
	**Univariate** ***P*****-value HR (95% CI)**		**Multivariate *P*-value**	**Univariate** ***P*****-value HR (95% CI)**		**Multivariate *P*-value**
**Age (years)**	0.462		NS	0.847		NS
≤46 years						
>46 years		/				/
**Gender**	0.804		NS	0.670		NS
Male						
Female		/			/	
**Smoking status**	0.208		NS	0.715		NS
Non-smoker						
Smoker		/			/	
**Alcohol abuse**	0.045		0.000	0.717		NS
Non-drinker		Reference				
Drinker		3.750 (1.909–7.367)			/	
**Tumor family history**	0.539		NS	0.858		NS
No						
Yes		/			/	
**Cranial nerve symptom**	0.029		NS	0.373		NS
No						
Yes		/			/	
**Baseline value of EBV-DNA**	0.138		NS	0.377		NS
≤2,000						
>2,000		/			/	
**Histological type**	0.910		NS	0.209		NS
WHO I/II						
WHO III		/			/	
**T stage**	0.327		NS	0.910		NS
1/2						
3/4		/			/	
**N stage**	0.070		NS	0.362		NS
0/1						
2/3		/			/	
**TNM stage**	0.041		NS	0.790		NS
I/II						
III/IV		/			/	
**Post-recurrence treatment options**	0.000		0.000	0.025		0.031
BST		Reference			Reference	
Salvage surgery		0.100 (0.042–0.238)			0.200 (0.065–0.619)	
Re-irradiation		0.159 (0.058–0.433)			0.266 (0.080–0.886)	
Chemotherapy		0.687 (0.309–1.528)			0.328 (0.100–1.071)	

**Table 9:** Univariate and multivariate analysis of post–recurrence prognostic factors of ELR and LLR in the locoregional recurrence group.

**Characteristic**	**ETR group**			**LTR group**		
	**Univariate** ***P*****-value HR (95% CI)**		**Multivariate *P*-value**	**Univariate** ***P*****-value HR (95% CI)**		**Multivariate *P*-value**
**Age (years)**	0.069		NS	0.296		NS
≤46 years						
>46 years		/			/	
**Gender**	0.982		NS	0.076		NS
Male						
Female		/			/	
**Smoking status**	0.284		NS	0.055		NS
Non-smoker						
Smoker		/			/	
**Alcohol abuse**	0.255		NS	0.489		NS
Non-drinker						
Drinker		/			/	
**Tumor family history**	0.144		NS	0.075		NS
No						
Yes		/			/	
**Cranial nerve symptom**	0.050		NS	0.563		NS
No						
Yes		/			/	
**Baseline value of EBV-DNA**	0.140		NS	0.963		NS
≤2,000						
>2,000		/			/	
**Histological type**	0.741		NS	0.056		NS
WHO I/II						
WHO III		/			/	
**T stage**	0.387	/	NS	0.006		0.014
1/2					Reference	
3/4					4.111 (1.337–12.635)	
**N stage**	0.020		0.008	0.472	/	NS
0/1		Reference				
2/3		2.216 (1.225–4.009)				
**TNM stage**	0.252		NS	0.028		NS
I/II						
III/IV		/			/	
**Post-recurrence treatment options**	0.002		0.007	0.000		0.000
BST		Reference			Reference	
Salvage surgery		0.238 (0.073–0.770)			0.277 (0.028–1.749)	
Re-irradiation		0.282 (0.090–0.885)			0.565 (0.071–1.476)	
Chemotherapy		0.666 (0.227–1.952)			0.736 (0.343–1.284)	

## Discussion

Here, we investigated the clinical characteristics and prognostic factors predicting OS and post-recurrence OS in NPC patients with ETR and LTR, ENR and LNR, and ELR and LLR. In this retrospective study, 409 (4.3%) developed purely local recurrence, 325 (3.4%) developed purely regional recurrence, and 182 (1.9%) developed locoregional recurrence, which is similar to the results of previous studies from other centers in China (12, 13); 192 patients (46.9%) developed early ETR, and 217 patients (53.1%) developed LTR, 183 patients (56.3%) developed ENR, and 142 patients (43.7%) developed LNR, while 87 patients (47.8%) developed ELR, and 95 patients (52.2%) developed LLR, which suggests that the incidence of early and late recurrence is nearly the same. The patients with LTR/LNR/LLR demonstrated significantly better OS than the patients with ETR/ENR/ELR, which is consistent with previous studies on renal cell carcinoma and intrahepatic cholangiocarcinoma (14, 15), while post-recurrence OS did not reach significance between the ETR and LTR, ENR and LNR, and ELR and LLR groups, which suggests that post-recurrence OS does not depend on the time of recurrence.

Multivariate Cox regression analysis revealed that age and gender were independent risk factors for OS with ETR, and the baseline value of EBV-DNA was an independent risk factor for OS with LTR; alcohol abuse and TNM stage were independent risk factors for OS with ENR, and no clinical characteristics were associated with OS with LTR, and N stage and TNM stage were independent risk factors for OS with ELR; and T stage was an independent risk factor for OS with LLR. In addition, multivariate Cox regression analysis revealed that post-recurrence treatment option was an independent risk factor of post-recurrence OS with ETR and LTR, while alcohol abuse and post-recurrence treatment option were independent risk factors of post-recurrence OS with ENR, and PRBST was associated with poorer post-recurrence OS with LNR. Meanwhile, N stage and post-recurrence treatment options were independent risk factors for post-recurrence OS with ELR, and T stage and post-recurrence treatment options were independent risk factors for post-recurrence OS with LLR. It has been suggested that patients with early initial T stage have a more favorable prognosis (16), which is consistent with the LLR patients in the present study. Post-recurrence treatment options including salvage surgery, re-irradiation, and chemotherapy are very important for NPC recurrence patients, which was shown that post-recurrence treatment options mentioned above have a better prognosis compared with PRBST.

There are various hypotheses for the occurrence of early and late recurrence. A probable hypothesis is the discrepancy of NPC tumor cell radiosensitivity. Recent studies have shown that apoptosis, DNA damage repair, a hypoxic microenvironment, and autophagy can be involved in regulating radiotherapy resistance (17–19), which results in the difference in early and late recurrence. A study from Japan shed light on the biological impact of DNA methylation status as a predictive biomarker of early recurrence in ovarian cancers (20). It has been suggested that the tumor dormancy-reactivation hypothesis might be applicable to NPC (21, 22). Furthermore, surgery for removing breast tumors may lead to the appearance of growth factors in the circulation in response to surgical wounding, which may terminate the dormancy of the tumor foci and result in accelerated recurrence (7, 23). Although the main treatment of NPC is radiotherapy instead of surgery, it may also give rise to the appearance and an increase of growth factors to result in the occurrence of recurrence. Therefore, it is reasonable to speculate that there might be intrinsic biological differences between patients with early and late recurrence, and this warrants further studies.

The definitions of ETR and LTR, ENR and LNR, and ELR and LLR were applied with 2 years as the cut-off point, which have also been proposed and proven in recent studies (8, 9, 24). Hence, the frequency and intensity of follow-up should be strengthened at the initial 2 years. However, the application of 2 years for differentiating early recurrence from late recurrence remains controversial, and several studies have used 5 years as another major demarcation point (14, 25, 26). We applied 5 years for differentiating early recurrence from late recurrence and we found that there are very few cases of early recurrence; the number imbalance between the two groups is likely to lead to statistical problems.

There are two new features in the present study that differ from previous reports. First, this study figured out the prognostic factors of OS and post-recurrence OS in NPC patients with ETR and LTR, ENR and LNR, and ELR and LLR in detail. Second, this study has a large sample size with long median-time follow-up, which might help oncologists predict patients' prognosis and design individualized follow-up strategies. Although our study yielded some unique results, certain limitations should be noted. First, this is a retrospective single-center study, which has inherent biases. Second, the study merely explores the impact of baseline clinical characteristics on post-recurrence OS rather than post-recurrence clinical characteristics, which are principal elements of post-recurrence OS. Third, some information was lacking because of the long follow-up duration. However, we believe that the present results are noteworthy and reliable because this is the only such large-cohort study to date.

## Conclusions

The present study shows that age and gender were independent risk factors of OS with ETR, and the baseline value of EBV-DNA was an independent risk factor of OS with LTR. Alcohol abuse and TNM stage were independent risk factors for OS with ENR, while N stage and TNM stage were independent risk factors of OS with ELR; and T stage was an independent risk factor for OS with LLR patients. In addition, post-recurrence treatment option was an independent risk factor of post-recurrence OS with ETR and LTR. Alcohol abuse and post-recurrence treatment option were independent risk factors of post-recurrence OS with ENR, and PRBST was associated with poorer post-recurrence OS with LNR. Meanwhile, N stage and post-recurrence treatment options were independent risk factors for post-recurrence OS with ELR, and T stage and post-recurrence treatment options were independent risk factors for post-recurrence OS with LLR. Patients with LTR/LNR/LLR demonstrate significantly better OS compared with patients with ETR/ENR/ELR, whereas post-recurrence OS is not significantly different between patients with ETR/ENR/ELR and LTR/LNR/LLR. Further studies are warranted to confirm our results.

## Data Availability Statement

The datasets generated for this study are available on request to the corresponding author.

## Ethics Statement

This study was performed according to the ethical principles of the Declaration of Helsinki, and the Sun Yat-sen University Cancer Center review board approved the study protocol. Written informed consent was obtained from all patients for their data to be used in clinical research without affecting their treatment options or violating their privacy.

## Author Contributions

FL, F-PC, Y-PC, and G-QZ conceived and designed the study. YC, X-JH, X-DH, and Z-QZ contributed cases data collection. W-HZ, XL, and YS analyzed the data. FL, F-PC, and G-QZ wrote the paper. All authors read and approved the final manuscript.

## Conflict of Interest

The authors declare that the research was conducted in the absence of any commercial or financial relationships that could be construed as a potential conflict of interest.

## Abbreviations

NPC: nasopharyngeal carcinoma
IMRT: intensity-modulated radiation therapy
ETR: early purely local recurrence
LTR: late purely local recurrence
ENR: early purely regional recurrence
LNR: late purely regional recurrence
ELR: early locoregional recurrence
LLR: late locoregional recurrence
OS: overall survival
WHO: World Health Organization
TNM: tumor-node-metastasis
MRI: magnetic resonance imaging
PET/CT: positron-emission tomography/computed tomography
UICC/AJCC: Union for International Cancer Control/American Joint Committee on Cancer
EBV: Epstein-Barr virus
HR: hazard ratio
CI: Confidence interval
RT: radiotherapy
Chemo: chemotherapy
PRBST: Post-recurrence best supportive treatment.
